# Methionine Promoted the Growth of Golden Pompano by Activating Intestinal Transport and TOR Signaling Pathway

**DOI:** 10.1155/anu/8592097

**Published:** 2025-06-04

**Authors:** Zhikang Song, Yuanyuan Zhou, Yingtao Li, Zhiwen Chen, Xingyuan Liu, Xinting Liu, Zhenzhu Sun, Chaoxia Ye

**Affiliations:** ^1^Guangzhou Key Laboratory of Subtropical Biodiversity and Biomonitoring, School of Life Science, South China Normal University, Guangzhou 510631, China; ^2^School of Biology and Agriculture, Shaoguan University, Shaoguan 512005, China

**Keywords:** amino acid transport, methionine, taurine synthesis, TOR pathway, *Trachinotus ovatus*

## Abstract

This experiment aimed to study the effects of methionine (Met) on growth performance, intestinal structure and transport, Met metabolism, and target of rapamycin (TOR) signaling pathway in golden pompano (*Trachinotus ovatus*). Fish (9.12 ± 0.03 g) were fed with six diets with 0.79%, 0.90%, 1.01%, 1.12%, 1.23%, and 1.34% DL-Met for 8 weeks. Our study showed that Met supplementation significantly increased fish growth, villus length, crypt depth, and the mRNA levels of intestinal amino acid transporters including asc-type amino acid transporter 1 (*asc-1*), sodium-dependent neutral amino acid transporter B (*asct2*), and cationic amino acid transporter1 (*cat1*) (*p* < 0.05). Liver histological analysis revealed that Met addition improved cell swelling, nuclear migration, and hepatic vacuolation. Appropriate Met supplementation significantly increased mRNA level of key genes (methionine adenosyl transferase [*mat*], cystathionine beta-synthase [*cbs*], cystathionine γ-lyase [*cse*], and cysteine dioxygenase [*cdo*]) involved in transmethylation, transsulfuration metabolism, and taurine synthesis pathways. The level of 1.12%–1.23%, 1.12%, and 1.23% Met significantly upregulated the mRNA expression of S-adenosyl methionine/target of rapamycin (*samtor*), regulatory-associated protein of TOR (*raptor*), and ribosomal protein s6 (*s6*), respectively. The protein levels of protein kinase B (AKT), TOR, p-TOR, S6K, p-S6K, and p-S6 increased firstly and then decreased with increasing dietary Met supplementation. In conclusion, Met supplementation may promote growth of golden pompano by improving intestinal structure and amino acid transport, increasing transmethylation and transsulfuration metabolism, and activating TOR signaling pathways through SAMTOR and AKT.

## 1. Introduction

Methionine (Met) is a building block of protein synthesis and participates in the formation of many metabolites, such as S-adenosylmethionine (SAM) and taurine [1]. In fish, the Met requirement ranges from 0.49% to 2.50% of feed (1.49% to 4.70% of feed protein) [2]. Digestion, absorption, and transport of nutrients take place primarily in the intestine. It has been reported that adequate dietary Met improved gut micromorphology of hybrid grouper [3] and increased intestinal villi height, microvilli height, and goblet cell number in turbot [4]. Amino acids enter the intestinal epithelium via amino acid transporters and are then transported to other tissues [5]. Met as a neutral amino acid can be transported by a variety of pathways, including sodium-dependent and non-sodium-dependent pathways. Met addition decreased the mRNA levels of SLC15A2, SLC7A8, and SLC38A2 in largemouth bass [6]. However, appropriate Met significantly increased the mRNA levels of B0AT1 and sodium-dependent neutral amino acid transporter B (ASCT2) in cobia intestine [7]. There are limited reports and conflicting results about the effect of Met on amino acid transporters in fish.

After absorption, Met is transported to the liver for metabolism. The major metabolic reactions of Met are transmethylation, transsulfuration, and remethylation. In the transmethylation pathway, Met is catalyzed by Met adenosine transferase (MAT2A) into SAM, which can be converted to S-adenosine homocysteine (SAH) and homocysteine (Hcy). In the remethylation pathway, Hcy can be remethylated to form Met with the catalysis of Met synthase (MS) or betaine homocysteine methyltransferase (BHMT). In the transsulfuration pathway, Hcy can be catalyzed by cystathionine beta-synthase (CBS) and cystathionine γ-lyase (CSE) to produce cysteine [8], which can be converted into taurine and glutathione by enzymes such as cysteine dioxygenase (CDO). Our previous experiment found that Met addition significantly increased taurine content and mRNA levels of *cdo*, suggesting that taurine can be synthesized by Met through transsulfuration in common carp [9]. On the contrary, Met supplementation decreased *cbs* mRNA expression and upregulated *ms* expression in turbot [4]. Effects of Met level on transmethylation, transsulfuration, and remethylation pathways remain poorly described in fish.

The target of rapamycin (TOR) signaling pathway senses growth factors and nutrients to regulate organism growth and homeostasis [10]. There are many studies showing that optimal Met intake promotes fish growth through the PI3K/AKT/TOR pathway regulated by growth hormone [2]. But how fish senses availability of Met? A recent study indicated that SAM, a Met metabolite, can dissociate S-adenosyl methionine/target of rapamycin (SAMTOR)–GATOR1 complex by binding directly to SAMTOR to activate TORC1 [11]. This led us to speculate that Met may also affect TORC1 activation through transmethylation metabolism and SAM content, thereby regulating growth.

The production of golden pompano (*Trachinotus ovatus*) reached 292,263 tons in 2023, and it is an important carnivorous marine aquaculture fish [12]. It has been reported that the Met requirement of *T. ovatus* was 1.06%–1.27% diet, corresponding to 2.46%–2.95% dietary protein according to weight gain (WG) and nitrogen retention efficiency [13], but how Met regulates the growth of golden pompano remains largely unknown. Our present experiment investigates the effects of Met on growth, intestinal structure and transport, Met metabolism, and TOR signaling pathway in golden pompano.

## 2. Materials and Methods

### 2.1. Experimental Diet

The dietary formulations are shown in Table 1. Six diets were made by adding 0%, 0.10%, 0.20%, 0.30%, 0.40%, and 0.50% DL-Met in the basal diet. The raw material was crushed, screened through 60 mesh, weighed precisely, and mixed thoroughly, and 30% water was added. Finally feed pellets with a diameter of 2.5 mm were made, air-dried, and stored at −20°C.

The experiment was performed in mesh cages in the pond. After 2 weeks of acclimatization, golden pompano (average weight 9.12 ± 0.03 g) were selected and distributed to 18 cages (1.0 m × 1.0 m × 1.8 m). There were 25 fish in each cage, and three cages were fed each feed. Except for typhoon days, golden pompano were fed twice a day. Feed intake (FI) was recorded once a week for 8 weeks. Water temperature, salinity, and pH were 32–34°C, 17–20‰, and 8.3–9.1, respectively. Nitrite was <0.01 mg/L, and ammonia nitrogen was <0.05 mg/L.

### 2.2. Sampling

After the end of experiment, golden pompano was starved for 24 h. Six fish per cage were used for whole fish composition analysis. Another five fish were selected from each cage, and blood was collected from the tail vein after eugenol anesthesia. Whole blood was centrifuged at 4000 rpm for 5 min to separate serum for biochemical index detection, and serum from five fish was pooled into one sample for testing. The visceral mass and liver weight of each fish were weighed separately, and the viscerosomatic index (VSI) and hepatosomatic index (HSI) were calculated. A portion of liver sample was immediately stored in RNA later overnight at 4°C for mRNA level analysis of key genes involved in Met metabolism and TOR signaling pathways, and the remaining portion was frozen in liquid nitrogen for subsequent western blotting analysis of key TOR signaling pathway proteins. Foregut sample was immediately stored in RNA later for mRNA expression analysis of amino acid transporters. Tissues were taken from fixed locations of liver and intestine, respectively, and fixed in 4% paraformaldehyde for sectionalization. Liver and intestine samples were delivered to Servicebio Co., Ltd., for slicing preparation. The intestinal and liver tissue morphology was photographed using a microscope.

### 2.3. Proximate Composition Analysis

The analysis of proximate composition was according to the standards established by AOAC in 2005 [14]. Moisture was analyzed by drying samples in oven at 105°C. Crude lipid of samples was analyzed using Soxhlet extraction instrument (Sweden, 2055 FOSS) with petroleum ether (boiling process 60–90°C). Crude protein was analyzed by Dumas method. Ash was analyzed by burning the sample in a muffle furnace (FO610C, Yamato Scientific Co., Ltd., Japan) after being fully carbonized.

### 2.4. Serum Biochemical Indexes

The activity of aspartate aminotransferase (AST) and alanine aminotransferase (ALT) and the levels of cholesterol (CHOL), low-density lipoprotein cholesterol (LDL-C), high-density lipoprotein cholesterol (HDL-C), glucose (Glu), triglyceride (TG), total protein (TP), and urea content were detected by the hospital of South China Normal University with automatic biochemical analyzer.

### 2.5. RNA Extraction and Q-PCR

The total RNA of the intestine and liver was extracted with Trizol (Vazyme, China). RNA integrity and concentration were analyzed by 1% agarose gel and NanoDrop 2000 spectrophotometer (NanoDrop Technologies, USA), respectively.

Q-PCR was used for relative quantitative analysis. The total reaction system consisted of 2 × SYBR Green Q-PCR Master Mix 10 μL, ddH_2_O 7.2 μL, cDNA 2 μL, and primer 0.4 μL each (Table 2). β-Actin was selected as the reference gene, and the 2^−∆∆CT^ method was used for gene expression calculation.

### 2.6. Western Blot Analysis

Livers were lysed and homogenized in a mixture containing RIPA buffer, PMSF (Beyotime Biotechnology), phosphatase inhibitor, and protease cocktails. The content of protein was analyzed by BCA protein concentration determination kit (Beyotime Biotechnology, P0010s). Samples were treated by SDS-PAGE and then transferred to PVDF membranes. After treated with 5% BSA, membranes were incubated with antibodies on ice overnight. Antibodies included Akt (Proteintech, 10176-2-p), phospho-Akt (Ser473) (Cell Signaling Technology, 9271), mTOR (Abways, CY3456), phospho-mTOR (Ser2448) (Cell Signaling Technology, 2971), S6K (Abways CY 5324), phospho-S6K (Thr389) (Cell Signaling Technology, 9205), and phospho-S6 ribosomal protein (Ser235/236) (Cell Signaling Technology, 4856) (1:2000; Elabscience E-AB-1003). Then membranes were incubated with secondary antibody (1:2000; Elabscience E-AB-1003). The image was developed by ECL chemiluminescence kit (Beyotime Biotechnology, P1008fs), and the gray value of the strip was quantified by ImageJ software.

### 2.7. Statistical Analysis

All data were analyzed by SPSS Statistics 26.0. One-way ANOVA and Duncan's multiple comparisons were applied to analyze all data. All data were analyzed using polynomial orthogonal contrasts to determine whether there were significant linear or quadratic responses. *p* < 0.05 was considered statistically significant. All data were shown as mean ± SEM.

## 3. Results

### 3.1. Growth Performance and Body Composition

As Met supplement increased, final body weight (FBW), WG, specific growth rate (SGR), and FI increased significantly (Table 3). Feed conversion ratio (FCR) and protein efficiency (PER) were not affected by Met supplementation. The survival rate (SR) ranged from 97.33% to 100% and not affected by Met levels. With increasing Met supplement, the condition factor (CF), VSI, and HSI first increased and then decreased, reaching the maximum in D3 (1.01%) and D4 (1.12%) groups, respectively.

Met supplement had no significant influence on protein and ash of the whole body (Table 4). When Met level was 1.12%, lipid was significantly higher, but moisture was significantly lower than other groups.

### 3.2. Serum Biochemical Indices

Table 5 showed that serum Glu and LDL-C levels first increased and then decreased as Met increased, reaching the maximum value in the D3 (1.01%) group. Serum HDL-C increased with increasing Met levels and was the highest in the 1.34% group. However, AST, ALT, CHOL, TP, and urea levels were not significantly affected by Met content.

### 3.3. Intestinal Morphology and Amino Acid Transporters

**Figure 1:**
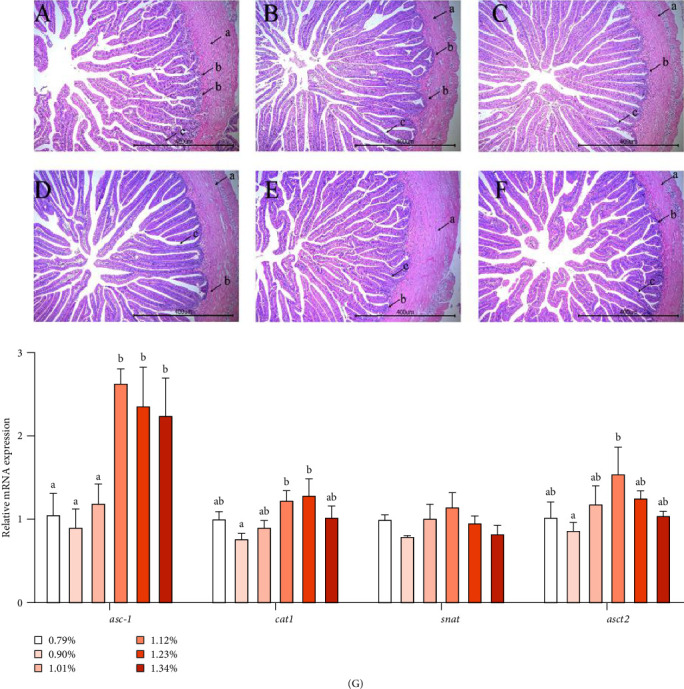
Effects of methionine on intestinal morphology and amino acid transporters of golden pompano (A–F), H&E staining (50×) of 0.79%, 0.90%, 1.01%, 1.12%, 1.23%, and 1.34% methionine groups. a, intestinal wall; b, crypt; c, villi. (G) Amino acid transporters. Bars of the same gene not sharing a common superscript letter are significantly different by Duncan's test (*p* < 0.05).

The morphology of the intestine is shown in Figure 1A–F. The length of intestinal villus became longer and more regular as dietary Met is increasing. The depth of crypt increased. The intestinal wall became thicker and more uniform.

As shown in Figure 1G, the mRNA expression of asc-type amino acid transporter 1 (*asc-1*) was significantly higher in the 1.12% to 1.34% Met groups than in the 0.79% to 1.01% Met groups. As Met increased, the mRNA level of cationic amino acid transporter1 (*cat1*) and *asct2* increased first and then decreased, reaching the maximum value in D5 (1.23%) and D4 (1.12%) groups, respectively. However, the mRNA expression of serotonin *N*-acetyltransferase (*snat*) was not affected by the level of Met.

### 3.4. Liver Morphology, Met Metabolism, and Taurine Synthesis

**Figure 2:**
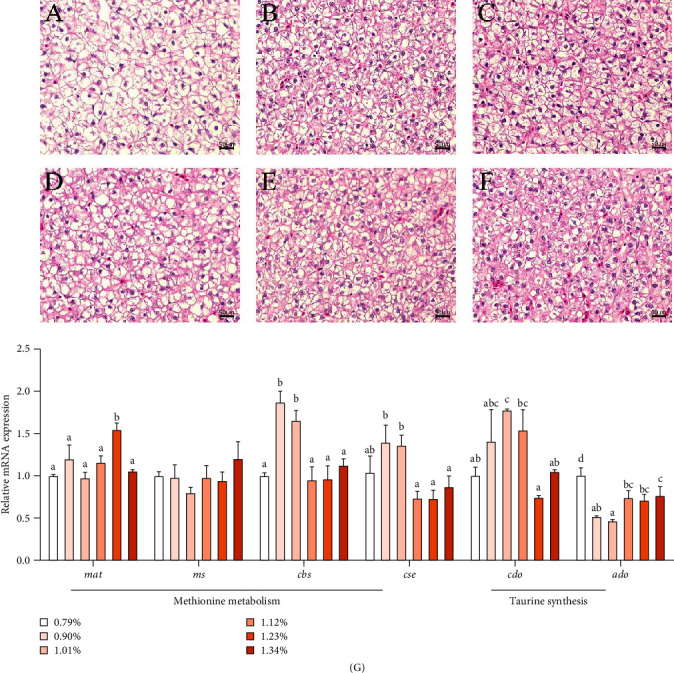
Effects of methionine on liver morphology and mRNA expression of methionine metabolism in golden pompano. (A–F) (H&E staining, 400 times) indicates that the methionine level is 0.79%, 0.90%, 1.01%, 1.12%, 1.23%, and 1.34%, respectively. (G) Methionine metabolism and taurine synthesis related genes. Bars of the same gene not sharing a common superscript letter are significantly different by Duncan's test (*p* < 0.05).

Liver tissue morphology (H&E staining) of golden pompano is illustrated in Figure 2. In the control group without Met addition, the nucleus moved to the edge of the cells, leading to the phenomenon of vacuolation, cell enlargement, and blurred cell boundaries. Met supplementation improved this phenomenon. The number and content of liver cells were increased in Met supplementation groups.

As shown in Figure 2G, the mRNA level of methionine adenosyl transferase [*mat*], a key gene of Met methyltransferase pathway, was the highest in 1.23% Met group. The mRNA expression of *cbs* and *cse* in 0.90% and 1.01% Met groups was higher than those in other groups. However, the mRNA level of *ms*, a key gene of Met remethylation metabolism pathway, was not significantly affected by Met. As Met increased, the mRNA level of *cdo*, a key gene for taurine synthesis in the liver, first increased and then decreased, reaching the maximum in the 1.01% group. However, 2-aminoethanethiol dioxygenase (*ado*) mRNA level decreased significantly with the Met supplementation, which was the lowest in 1.01% group.

### 3.5. MRNA Expression of TOR Signaling Pathway

**Figure 3:**
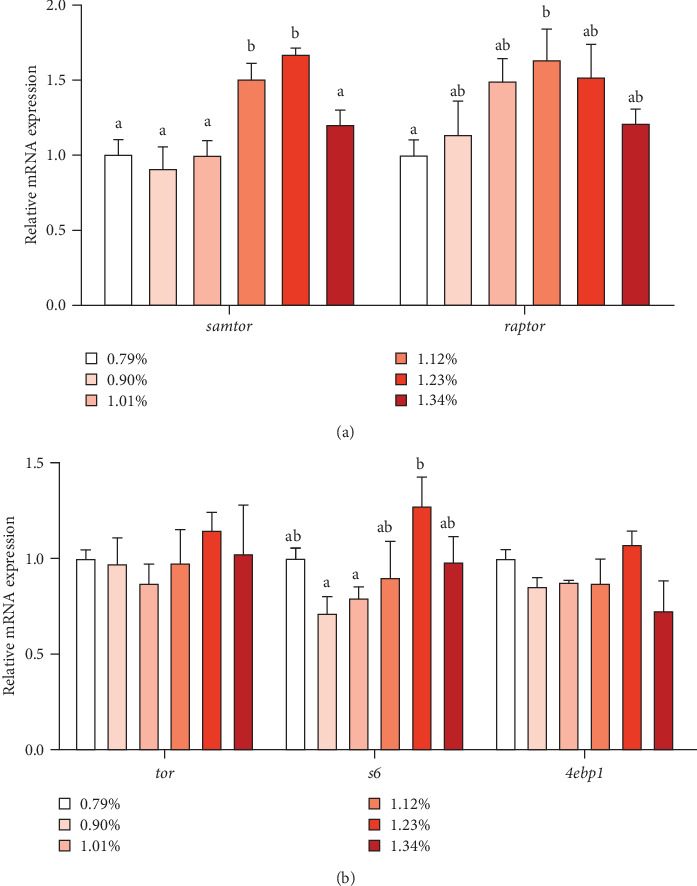
Effects of dietary methionine levels on the mRNA expression of genes involved in TOR pathway in the liver of *Trachinotus ovatus*. The value (three replicates) is expressed as mean ± standard error. (A) Upstream related genes of mTOR. (B) mTOR signaling pathway related genes. Bars of the same gene not sharing a common superscript letter are significantly different by Duncan's test (*p* < 0.05).

As shown in Figure 3, the mRNA expression of *samtor* and regulatory-associated protein of TOR (*raptor*) increased first and then decreased with increasing Met, reaching the maximum at 1.23% and 1.12% Met levels, respectively. As Met increased, protein synthesis-related gene ribosomal protein s6 (*s6*) first increased and then decreased, reaching the maximum in D5 (1.23%) group. However, the mRNA level of *tor* and eukaryotic translation initiation factor 4E-binding protein 1 (*4ebp1*) was not affected by different treatments.

### 3.6. Protein Expression of TOR Signaling Pathway

**Figure 4:**
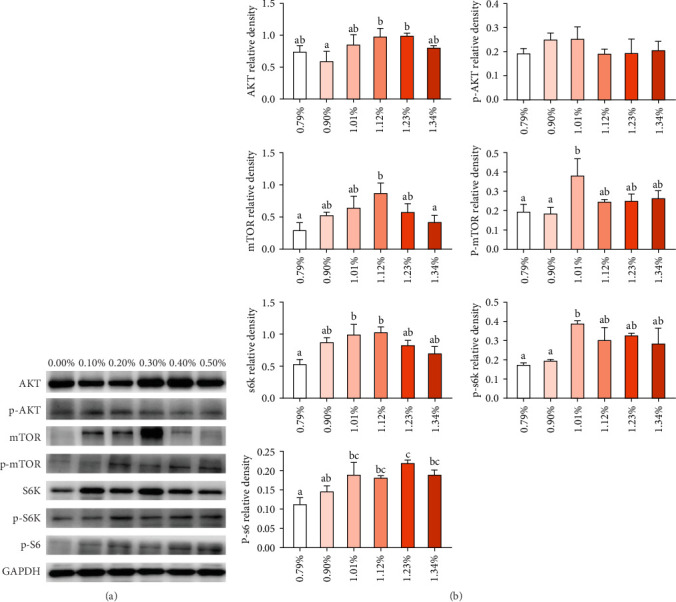
Effects of methionine on TOR key protein expression in the liver of *Trachinotus ovatus*. (A) Western blot results. (B) The statistical calculation of grayscale value of strips is based on the relative ratio of protein/internal reference. The value (three replicates) is expressed as mean ± standard error. Bars not sharing a common superscript letter are significantly different by Duncan's test (*p* < 0.05).

As showed in Figure 4, the protein expression of AKT increased first and then decreased, reaching the maximum value in 1.12% and 1.23% Met group. However, protein expression of p-AKT was not significantly affected by dietary Met supplement. Met addition increased protein expression of TOR, p-TOR, S6K, p-S6K, and p-S6. Protein levels of TOR, p-TOR, S6K, and p-S6K showed similar trend with Akt, reaching the maximum value in 1.12% and 1.01% Met groups. The protein expression of p-S6 kept increasing as Met supplement increased up to 1.23%.

## 4. Discussion

Due to the important role of Met in growth and metabolism, adding Met to low fish meal feed to balance amino acids has been widely used in the aquatic industry. As reviewed by Wang [2], appropriate Met in the diet can promote growth of most fish, but different fish respond differently to excessive Met. In this experiment, 1.12%–1.34% dietary Met supplementation significantly increased FBW, WG, SGR, and FI, similar to the results of another experiment in golden pompano [13] and silver pompano [15]. The effects of Met supplement on whole body composition are not very consistent in different experiments. In this experiment, dietary supplementation of 1.12% Met increased whole-body crude lipid and decreased moisture. The serum levels of LDL-C also first increased and then decreased, reaching the maximum value in 1.01% Met group, indicating that appropriate Met supplementation was beneficial to increase lipid deposition. Our results were similar with the study in cobia, which showed that liver lipid, total CHOL, and TG increased first and then decreased with increasing Met supplement [16]. Our study showed that serum Glu increased when Met was 0.90% and 1.01%, which was similar to the trends of serum Glu content in Chinese sucker [17], cobia [18], and grouper [19]. We speculate that Met can improve the deposition of lipid and Glu in fish, which is one of the factors that promotes fish growth.

The development of intestinal mucosal structure will directly affect digestion and absorption of nutrients [20]. Our present experiment showed that adding Met increased villus length and crypt depth, which is consistent with the findings in grouper [3] and turbot [4], indicating that Met contents affect the development of intestinal structure. Protein in feed is digested into polypeptide and amino acid under the action of digestive enzyme and then enters intestinal epithelial cells through amino acid transporter and peptide transporter. Addition of 0.60% Met significantly increased WG and intestinal *asct2* expression of cobia [7]. In our experiment, the expression of *asc-1* significantly increased when Met was ≥1.12%. As Met increased, the mRNA expression of *asct2* and *cat1* increased first and then decreased, while *snat* expression was not significantly affected by Met. ASCT is located in the apical membrane and responsible for amino acid absorption including Met into intestinal cells, while *asc-1* is an amino acid transporter located in the basement membrane, which is mainly responsible for the transport of amino acids into capillaries, portal vein, and systemic circulation, which promote the transport of amino acids. Therefore, this study may suggest that Met can improve the development of intestinal mucosal structure and promote the mRNA level of amino acid transporters, thereby increasing intracellular amino acid content and promoting protein synthesis.

The liver is the main organ of Met metabolism, and liver cell damage will affect the metabolic response of liver cells. Hepatic H&E staining showed that Met addition alleviated the problems of hepatocyte nuclear migration, swelling, and cell boundary blurring caused by Met deficiency, indicating that Met could reduce hepatocyte injury and facilitate the metabolic response of liver cells. Met is converted to SAM in response to MAT and ATP. On the one hand, SAM is a major methyl donor for intracellular methylation of hormones, amines, and DNA, promoting cell growth and differentiation [21, 22]; on the other hand, SAM is converted to Hcy, which can be converted to cysteine by sequential catalysis of CBS and CSE in the transsulfuration pathway [23]. In this experiment, appropriate dietary Met supplementation significantly improved mRNA expression of transmethylation pathway key gene (*mat*) and transsulfuration pathway key genes (*cbs* and *cse*) but had no effect on the major genes of remethylation pathway (*ms*), indicating that appropriate dietary Met supplementation could enhance transmethylation and transsulfuration. Cysteine can form disulfide bonds, which gives it an important role in protein structure and protein folding pathways. Our results suggest that Met supplementation may provide more cysteine for protein synthesis and promote fish growth. Another destination for cysteine is to synthesis taurine [24]. Taurine is important for metabolic regulation of golden pompano [25]. In our experiment, the mRNA level of *cdo*, a key gene in cysteine taurine synthesis pathway, was increased in appropriate Met added groups. The results were similar to our previous study in common carp, in which Met addition upregulated *cdo* expression and taurine content in serum and muscle [9]. An alternative pathway for taurine synthesis depends on ADO. Our present study and previous study [9] found that low Met supplementation decreased *ado* expression, but high Met supplementation resulted in higher *ado* expression than low Met supplementation. This phenomenon is interesting, and it is possible that fish can regulate and balance the two taurine synthesis pathways under different Met concentrations. In a word, appropriate Met supplementation can promote transmethylation and transsulfuration metabolism, thereby providing SAM, cysteine, taurine, and other substances for fish growth.

TOR signaling pathway is the main pathway regulating intracellular protein synthesis [26]. It has been reported that SAM, a Met metabolic derivative, is an effective regulator of mTORC1 activity, which binds to a protein called SAMTOR to activate mTORC1 activity [11]. SAM rather than Met levels appear to be sensed to monitor Met metabolic status in most known pathways. The scaffold protein RAPTOR is the main protein of mTORC1, which regulates mTORC1 assembly and recruit kinase substrate. RAPTOR is important in determining the subcellular localization of mTORC1 and detecting amino acids [10]. Our study found the mRNA levels of *samtor* and *raptor* significantly increased when Met was up to 1.12%, and the protein expression of TOR and p-TOR also reached the maximum value in 1.12% and 1.01% Met groups. Our results indicate that appropriate Met supplementation may increase SAM production, enhance SAM sensing, and activate TORC1. Studies have shown that activated mTORC1 directly phosphorylates 4EBP1 and S6K1 [10]. 4EBP1 is a regulatory factor in translation initiation. The phosphorylation of S6K1 can phosphorylate the S6 protein of ribosome and promote the initiation and extension of translation. In this experiment, S6K, p-S6K, and P-S6 protein expression and *s6* mRNA expression were significantly upregulated, indicating that dietary Met supplementation may promote growth through SAMTOR/TOR signaling pathway. Meanwhile, our experiment showed that AKT protein expression also increased significantly in the appropriate Met supplementation groups, suggesting that optimal Met also promotes growth in fish by the PI3K/Akt/TOR pathway, similar with studies in humpback grouper [27], cobia [7, 16, 28], and rainbow trout [29]. The signaling pathways underlying the growth-promoting effects of Met are likely to be multifaceted and require further in-depth investigation.

## 5. Conclusions

Our study showed that appropriate Met supplementation improved growth, intestinal structure, and amino acid transporter expression, thereby increasing more Met for protein synthesis and Met metabolism. In addition, Met supplementation improved liver structure, increased transmethylation and transsulfuration metabolism, and activated TOR pathway by enhancing AKT protein expression and SAMTOR gene expression.

## Figures and Tables

**Table 1:** Ingredient and proximate composition of diets used in this study (% as is).

Ingredients	D1 (0.79%)	D2 (0.90%)	D3 (1.01%)	D4 (1.12%)	D5 (1.23%)	D6 (1.34%)
Fish meal^a^	22.00	22.00	22.00	22.00	22.00	22.00
Soybean^b^	16.00	16.00	16.00	16.00	16.00	16.00
Krill meal^c^	3.00	3.00	3.00	3.00	3.00	3.00
Chicken meal^d^	3.00	3.00	3.00	3.00	3.00	3.00
Peanut meal^e^	10.00	10.00	10.00	10.00	10.00	10.00
Fish oil	5.09	5.09	5.09	5.09	5.09	5.09
Phospholipid	2.00	2.00	2.00	2.00	2.00	2.00
Wheat flour	25.00	25.00	25.00	25.00	25.00	25.00
Ca(H_2_PO_4_)_2_	2.00	2.00	2.00	2.00	2.00	2.00
Choline chloride (60%)	0.50	0.50	0.50	0.50	0.50	0.50
Mineral and vitamin mix^f^	2.00	2.00	2.00	2.00	2.00	2.00
Cellulose	5.28	5.28	5.28	5.28	5.28	5.28
Y_2_O_3_	0.10	0.10	0.10	0.10	0.10	0.10
Vitamin C	0.50	0.50	0.50	0.50	0.50	0.50
DL-Met^g^	0.00	0.10	0.20	0.30	0.40	0.50
Gly	0.50	0.40	0.30	0.20	0.10	0.00
Cys	0.07	0.07	0.07	0.07	0.07	0.07
Amino acid premix^h^	3.05	3.05	3.05	3.05	3.05	3.05
Proximate composition (% dry weight)						
Met level	0.79	0.90	1.01	1.12	1.23	1.34
Crude protein	42.31	42.28	42.15	42.46	42.04	42.09
Crude lipid	11.74	11.54	11.43	12.25	11.87	11.89

^a^Fish meal: imported from Peru.

^b^Soybean meal: Guangzhou Dachuan Feed Co., Ltd.

^c^Kill meal: Shandong Bodelong Group Co., Ltd. Weihai city, Shandong province.

^d^Chicken meal: imported from the United States.

^e^Peanut meal: Qingdao Jiali Peanut Oil Co., Ltd.

^f^Mineral and vitamin mix (mg/kg): vitamin A, ≥250,000 IU; vitamin D, 35,000–200,000 IU; vitamin E, ≥4000 mg; vitamin K_3_, ≥1100 mg; microbial B_1_, ≥820 mg; vitamin B_2_, ≥800 mg; vitamin B_6_, ≥2500 mg; vitamin B_12_, ≥5 mg; vitamin C, ≥12,000 mg; D-calcium pantothenate, ≥2500 mg; nicotinamide, ≥6000 mg; folic acid, ≥500 mg; D-biotin, ≥10 mg; inositol, ≥3000 mg; magnesium, ≥5000 mg; zinc, 2360–15,000 mg; manganese, 630–15,000 mg; copper, 170–2500 mg; iron, 4500–75,000 mg; cobalt, 130–200 mg; iodine, 80–2000 mg; selenium, 26–50 mg; moisture, ≤10%; purchased from Guangdong Hyint Biotechnology Group Co., Ltd.

^g^DL-Met: imported from Evonik Industries AG.

^h^Amino acid premix: Lys, 0.55%; Arg, 0.40%; Leu, 0.53%; Thr, 0.30%; Trp, 0.09%; Ile, 0.31%; Val, 0.36%; His, 0.21%; Phe, 0.30%.

**Table 2:** Primers used for real-time PCR.

Gene	Quantitative PCR primers, forward/reverse (5′ to 3′)	
	Forward primer (5′ to 3′)	Reverse primer (5′ to 3′)
*asc-1*	CACTGCCACCATAGTCATCCTG	CATACCCAAGCCACAAACCA
*snat*	TGGTGATGACTGTTCGGTTAGC	CAAAATCGCAGCCTTACGG
*asct2*	GCTGCCCGCCATCTACTTT	CGCCGTTATTCTCCTCCACA
*cat*	CCTGCCATTGTGCTGTCTTT	CTTCCCCACCAACTCGTCA
*mat*	CAGATGAGGATGCTGTCAAAGG	CGGTGCTGATGTAGAGGAAGAA
*cbs*	CAACCCACTGGCTCACTACG	TACCCGATGCCCTCTACCTC
*ms*	TGGGCGAACTTTGTCTGGT	TGCCTCAATGAATGGTCTCAT
*cdo*	CATTGGCCTGCATCGTGT	TGGTGACCTTCAGTTGTTCTCT
*ado*	CGACCTGAAAATTGCACCC	ACGCTCACCTTCCCATAGA
*cse*	CGATTTCCCTTTCTACCACA	ACAGCCTTCTCAAGACAGTTTC
*samtor*	TTGGATGGCGGGATGAAA	R: CCTGTATGGCTGGGCACTT
*raptor*	TCGCAGACCAGAAGAACCC	CTCCCAGTCCACAACCATACC
*mtor*	GAGCACCAGGGTCTTATGAGCCA	CTTCAGGGTTGTCAGCGGATTGT
*s6*	TGGGAGATGAGTGGAAGGG	CGGGGACGGTAGCAGGAGT
*4ebp1*	ACACCCCAGCAGGAACTTT	GTGACCATCAACGACGCAG
*β-actin*	TACGAGCTGCCTGACGGACA	GGCTGTGATCTCCTTCTGC

Abbreviations: *4ebp1*, eukaryotic translation initiation factor 4E-binding protein 1; *ado*, 2-aminoethanethiol dioxygenase; *asc-1*, asc-type amino acid transporter 1; *asct2*, sodium-dependent neutral amino acid transporter B; *cat1*, cationic amino acid transporter1; *cbs*, cystathionine beta-synthase; *cdo*, cysteine dioxygenase; *cse*, cystathionine *γ*-lyase; *mat*, methionine adenosyl transferase; *ms*, methionine synthase or 5-methyltetrahydrofolate-homocysteine methyltransferase; *mtor*, mechanistic target of rapamycin; *raptor*, regulatory-associated protein of TOR; *s6*, ribosomal protein s6; *samtor*, S-adenosyl methionine/target of rapamycin; *snat*, serotonin *N*-acetyltransferase.

**Table 3:** Growth performance and feed utilization of golden pompano fed with different levels of methionine^1^.

	D1 (0.79%)	D2 (0.90%)	D3 (1.01%)	D4 (1.12%)	D5 (1.23%)	D6 (1.34%)	*p*-Value		
							ANOVA	Linear	Quadratic
In^2^	9.10 ± 0.01	9.12 ± 0.01	9.15 ± 0.03	9.10 ± 0.03	9.16 ± 0.04	9.09 ± 0.04	0.438	0.941	0.213
FBW^3^	82.87 ± 2.30^a^	86.59 ± 2.06^a,b^	86.85 ± 2.75^a,b^	93.62 ± 3.45^b,c^	93.22 ± 1.99^b,c^	95.40 ± 1.47^c^	0.018	0.001	0.649
WG^4^	810.84 ± 24.39^a^	849.49 ± 21.54^a,b^	849.05 ± 32.72^a,b^	928.79 ± 35.96^b,c^	917.88 ± 22.62^b,c^	949.88 ± 19.21^c^	0.019	0.001	0.765
SGR^5^	4.33 ± 0.05^a^	4.41 ± 0.04^a,b^	4.41 ± 0.07^a,b^	4.57 ± 0.07^b,c^	4.55 ± 0.04^b,c^	4.61 ± 0.04^c^	0.019	0.001	0.730
FCR^6^	1.37 ± 0.03	1.40 ± 0.05	1.41 ± 0.04	1.36 ± 0.02	1.36 ± 0.02	1.34 ± 0.01	0.573	0.257	0.333
FI^7^	100.62 ± 2.01^a^	106.34 ± 4.78^a,b^	109.26 ± 2.43^a,b^	114.43 ± 3.48^b^	113.65 ± 3.88^b^	115.78 ± 2.91^b^	0.048	0.003	0.311
PER^8^	1.73 ± 0.04	1.69 ± 0.06	1.68 ± 0.05	1.74 ± 0.02	1.76 ± 0.02	1.77 ± 0.02	0.555	0.194	0.290
SR^9^	100.00 ± 0.00	97.33 ± 1.33	98.67 ± 1.33	100.00 ± 0.00	97.33 ± 1.33	100.00 ± 0.00	0.162	0.869	0.241
CF^10^	3.57 ± 0.11	3.72 ± 0.04	3.84 ± 0.06	3.75 ± 0.08	3.64 ± 0.07	3.52 ± 0.10	0.134	0.417	0.011
HSI^11^	1.11 ± 0.05	1.08 ± 0.04	1.21 ± 0.03	1.22 ± 0.06	1.12 ± 0.04	1.02 ± 0.04	0.062	0.351	0.011
VSI^12^	5.98 ± 0.09	6.05 ± 0.08	6.39 ± 0.10	6.29 ± 0.14	6.22 ± 0.21	6.11 ± 0.09	0.250	0.351	0.046

^1^Within a row, values not sharing a common superscript letter are significantly different (*p* < 0.05).

^2^In: initial body weight, g/fish.

^3^FBW: final body weight, g/fish.

^4^WG (weight gain, %) = 100 × (final body weight – initial body weight)/initial body weight.

^5^SGR (specific growth rate, %/day) = 100 × (ln final body weight – ln initial body weight)/days.

^6^FCR (feed conversion ratio) = dry feed intake/wet weight gain.

^7^FI (feed intake, g/fish) = dry feed intake/final fish number.

^8^PER (protein efficiency ratio) = (total final body weight – total initial body weight)/(dry feed intake × protein content in the feed).

^9^SR (survival rate, %) = 100 × (final fish number/initial fish number).

^10^CF (condition factor, g/cm^3^) = 100 × weight/body length^3^.

^11^HSI (hepatosomatic index, %) = 100 × liver wet weight/body wet weight.

^12^VSI (viscerosomatic index, %) = 100 × visceral wet weight/body wet weight.

**Table 4:** Effects of methionine levels on whole-body composition of golden pompano (%, wet weight)^1^.

	D1 (0.79%)	D2 (0.90%)	D3 (1.01%)	D4 (1.12%)	D5 (1.23%)	D6 (1.34%)	*p*-Value		
							ANOVA	Linear	Quadratic
Moisture	67.68 ± 0.25^c^	67.73 ± 0.05^c^	67.44 ± 0.39^b,c^	66.26 ± 0.07^a^	66.88 ± 0.00^a,b^	67.7 ± 0.18^c^	0.012	0.156	0.013
Protein	16.89 ± 0.29	17.32 ± 0.19	17.16 ± 0.08	17.40 ± 0.06	17.39 ± 0.08	17.31 ± 0.20	0.767	0.173	0.722
Lipid	12.05 ± 0.23^a^	11.68 ± 0.05^a^	11.82 ± 0.24^a^	12.79 ± 0.11^b^	12.10 ± 0.11^a^	11.58 ± 0.24^a^	0.031	0.631	0.314
Ash	3.85 ± 0.03	3.93 ± 0.03	3.88 ± 0.02	3.88 ± 0.03	3.89 ± 0.03	3.91 ± 0.06	0.714	0.573	0.828

^1^Within a row, values not sharing a common superscript letter are significantly different (*p* < 0.05).

**Table 5:** Effects of dietary methionine levels on serum biochemical indices of golden pompano^1^.

	D1 (0.79%)	D2 (0.90%)	D3 (1.01%)	D4 (1.12%)	D5 (1.23%)	D6 (1.34%)	*p*-Value		
							ANOVA	Linear	Quadratic
ALT (U/L)	4.33 ± 0.33	5.67 ± 0.67	4.67 ± 0.67	3.33 ± 0.88	5.33 ± 0.33	5.00 ± 1.00	0.285	0.866	0.575
AST (U/L)	26.00 ± 1.53	40.33 ± 4.48	27.33 ± 5.36	33.33 ± 10.73	41.33 ± 11.14	30.33 ± 4.91	0.566	0.622	0.532
CHOL (mmol/L)	4.07 ± 0.13	4.41 ± 0.07	4.66 ± 0.22	4.60 ± 0.38	4.49 ± 0.20	4.61 ± 0.26	0.516	0.164	0.252
GLU (mmol/L)	20.20 ± 0.76^a,b^	22.18 ± 1.51^b^	22.66 ± 1.89^b^	17.95 ± 0.60^a^	20.18 ± 0.09^a,b^	18.06 ± 0.87^a^	0.049	0.041	0.213
HDL-C (mmol/L)	2.83 ± 0.10	3.00 ± 0.07	3.01 ± 0.05	3.07 ± 0.15	3.02 ± 0.14	3.23 ± 0.10	0.265	0.034	0.955
LDL-C (mmol/L)	0.37 ± 0.02	0.47 ± 0.04	0.59 ± 0.07	0.56 ± 0.10	0.45 ± 0.02	0.44 ± 0.06	0.164	0.564	0.018
TG (mmol/L)	1.93 ± 0.09	2.04 ± 0.10	2.23 ± 0.20	1.96 ± 0.06	2.11 ± 0.11	1.82 ± 0.10	0.268	0.547	0.070
TP (g/L)	35.47 ± 0.09	38.93 ± 0.88	38.37 ± 2.35	36.60 ± 0.60	38.73 ± 1.17	37.03 ± 3.66	0.748	0.735	0.402
Urea (mmol/L)	0.64 ± 0.02	0.55 ± 0.04	0.54 ± 0.03	0.53 ± 0.02	0.56 ± 0.10	0.56 ± 0.06	0.709	0.406	0.211

^1^Within a row, values not sharing a common superscript letter are significantly different (*p* < 0.05).

## Data Availability

The data are available from the corresponding author upon reasonable request.

## References

[1] Mai K., Xue M., He G., Xie S. Q., Kaushik S. J., Hardy R. W., Kaushik S. J. (2022). Protein and Amino Acids. *Fish Nutrition*.

[2] Wang L., Gao C., Wang B., Wang C., Sagada G., Yan Y. (2023). Methionine in Fish Health and Nutrition: Potential Mechanisms, Affecting Factors, and Future Perspectives. *Aquaculture*.

[3] Li X., Mu W., Wu X. (2020). The Optimum Methionine Requirement in Diets of Juvenile Hybrid Grouper (*Epinephelus fuscoguttatus*♀ × *Epinephelus lanceolatus*♂): Effects on Survival, Growth Performance, Gut Micromorphology and Immunity. *Aquaculture*.

[4] Gao Z., Wang X., Zhou H., Mai K., He G. (2019). Effect of Dietary Methionine Levels on Growth Performance, Amino Acid Metabolism and Intestinal Homeostasis in Turbot (*Scophthalmus maximus* L.). *Aquaculture*.

[5] Bröer S. (2008). Amino Acid Transport Across Mammalian Intestinal and Renal Epithelia. *Physiological Reviews*.

[6] Wang W., Yang P., He C., Chi S., Song F. (2021). Effects of Dietary Methionine on Growth Performance and Metabolism Through Modulating Nutrient-Related Pathways in Largemouth Bass (*Micropterus salmoides*). *Aquaculture Reports*.

[7] He Y., Chi S., Tan B. (2019). DL-Methionine Supplementation in a Low-Fishmeal Diet Affects the TOR/S6K Pathway by Stimulating ASCT2 Amino Acid Transporter and Insulin-Like Growth Factor-I in the Dorsal Muscle of Juvenile Cobia (*Rachycentron canadum*). *British Journal of Nutrition*.

[8] Lauinger L., Kaiser P. (2021). Sensing and Signaling of Methionine Metabolism. *Metabolites*.

[9] Zhou Y., He J., Su N. (2021). Effects of DL-Methionine and a Methionine Hydroxy Analogue (MHA-Ca) on Growth, Amino Acid Profiles and the Expression of Genes Related to Taurine and Protein Synthesis in Common Carp (*Cyprinus carpio*). *Aquaculture*.

[10] Laplante M., Sabatini D. M. (2012). mTOR Signaling in Growth Control and Disease. *Cell*.

[11] Gu X., Orozco J. M., Saxton R. A., Condon K. J., Liu G. Y., Krawczyk P. A. (2017). Scaria, SAMTOR Is an S-Adenosylmethionine Sensor for the mTORC1 Pathway. *Science*.

[12] Bureau of Fisheries, M.O.A (2024). *China Fishery Statistical Yearbook*.

[13] Niu J., Du Q., Lin H. Z. (2013). Quantitative Dietary Methionine Requirement of Juvenile Golden Pompano *Trachinotus ovatus* at a Constant Dietary Cystine Level. *Aquaculture Nutrition*.

[14] AOAC (2005). *Official Methods of Analysis of AOAC INTERNATIONAL*.

[15] Ebeneezar S., Vijayagopal P., Srivastava P. P. (2020). Optimum Dietary Methionine Requirement of Juvenile Silver Pompano, *Trachinotus blochii* (Lacepede, 1801). *Animal Feed Science and Technology*.

[16] Wang Z., Mai K., Xu W., Zhang Y., Liu Y., Ai Q. (2016). Dietary Methionine Level Influences Growth and Lipid Metabolism via GCN2 Pathway in Cobia (*Rachycentron canadum*). *Aquaculture*.

[17] Chu Z. J., Gong Y., Lin Y. C. (2014). Optimal Dietary Methionine Requirement of Juvenile Chinese Sucker, *Myxocyprinus asiaticus*. *Aquaculture Nutrition*.

[18] Zhou Q.-C., Wu Z.-H., Tan B.-P., Chi S.-Y., Yang Q.-H. (2006). Optimal Dietary Methionine Requirement for Juvenile Cobia (*Rachycentron canadum*). *Aquaculture*.

[19] Luo Z., Liu Y., Mai K. (2005). Dietary L-Methionine Requirement of Juvenile Grouper *Epinephelus coioides* at a Constant Dietary Cystine Level. *Aquaculture*.

[20] Berge G. E., Goodman M., Espe M., Lied E. (2004). Intestinal Absorption of Amino Acids in Fish: Kinetics and Interaction of the in Vitro Uptake of l-Methionine in Atlantic Salmon (*Salmo salar* L.). *Aquaculture*.

[21] Stipanuk M. H. (2004). Sulfur Amino Acid Metabolism: Pathways for Production and Removal of Homocysteine and Cysteine. *Annual Review of Nutrition*.

[22] Teodoro B. (2002). S-Adenosyl-l-Methionine (SAMe): From the Bench to the Bedside—molecular Basis of a Pleiotrophic Molecule. *American Journal of Clinical Nutrition*.

[23] Brosnan J. T., Brosnan M. E. (2006). The Sulfur-Containing Amino Acids: An Overview. *The Journal of Nutrition*.

[24] Salze G. P., Davis D. A. (2015). Taurine: A Critical Nutrient for Future Fish Feeds. *Aquaculture*.

[25] Ma Q., Guo H., Zhu K. (2021). Dietary Taurine Intake Affects Growth and Taurine Synthesis Regulation in Golden Pompano, *Trachinotus ovatus* (Linnaeus 1758). *Aquaculture*.

[26] Liu G. Y., Sabatini D. M. (2020). mTOR at the Nexus of Nutrition, Growth, Ageing and Disease. *Nature Reviews Molecular Cell Biology*.

[27] Irm M., Mu W., Xiaoyi W., Geng L., Zhou Z. (2021). The Optimum Dietary Methionine Requirement of Juvenile Humpback Grouper (*Cromileptes altivelis*): Effects on Growth, Micromorphology, Protein and Lipid Metabolism. *Amino Acids*.

[28] Chi S., He Y., Zhu Y. (2019). Dietary Methionine Affects Growth and the Expression of Key Genes Involved in Hepatic Lipogenesis and Glucose Metabolism in Cobia (*Rachycentron canadum*). *Aquaculture Nutrition*.

[29] Rolland M., Dalsgaard J., Holm J., Gómez-Requeni P., Skov P. V. (2015). Dietary Methionine Level Affects Growth Performance and Hepatic Gene Expression of GH-IGF System and Protein Turnover Regulators in Rainbow Trout (*Oncorhynchus mykiss*) Fed Plant Protein-Based Diets. *Comparative Biochemistry and Physiology Part B: Biochemistry and Molecular Biology*.

[30] Song Z., Zhou Y., Li Y. (2025). Methionine Promoted the Growth of Golden Pompano by Activating Intestinal Transport and TOR Signaling Pathway. https://papers.ssrn.com/sol3/papers.cfm?abstract_id=5074228.

